# Supplementary material for: Long-Term Physical Activity Mitigates Inflammaging Progression in Older Adults Amidst the COVID-19 Pandemic
Source: Int J Environ Res Public Health. 2024 Oct 27;21(11):1425. doi: 10.3390/ijerph21111425 (PMC11593455; doi:10.3390/ijerph21111425)
Supplement: Supplementary file 1 [file ijerph-21-01425-s001.zip › ijerph-3218104-supplementary.pdf]

Table S1. Results [median and interquartile range (X_25-75)] concerning of systemic cytokine concentration (IL-6, IL-8, IL-10, IL-12p70, TNF-α, and IFN-γ) and also the ratio between IL-6/IL-10, IL-8/IL-10, IL-12p70/IL-10, TNF-α/IL-10, IFN-γ/IL-10. In addition, the data obtained in 2019, 2021 and 2022.

Variables	Volunteer Groups					
	Older women (n=18)			Older men (n=7)		
	2019	2021	2022	2019	2021	2022
IL-6 (pg/mL)	7.63 (6.46-10.59)	4.48 (3.98-5.60)	5.81 (3.89-7.09)	7.17 (6.04-7.90)	6.88 (4.42-7.76)	6.53 (5.13-12.42)
IL-8 (pg/mL)	237.2 (34.52-465.0)	238.9 (175.6-326.7)	126.6 (102-141.7)	200.2 (180.0-212.6)	313.8 (145.5-367.8)	179.6 (126.9-261.9)
IL-10 (pg/mL)	7.27 (5.66-9.16)	2.80 (2.36-3.11)	4.82 (3.40-8.07)	5.93 (5.28-7.27)	2.08 (1.85-4.24)	4.48 (3.96-4.56)
IL-12p70 (pg/mL)	7.59 (6.11-10.17)	2.65 (1.37-3.98)	11.31 (0.1-33.83)	6.39 (6.04-10.91)	2.92 (1.31-5.92)	9.69 (0.01-23.17)
TNF-α (pg/mL)	9.72 (7.26-20.07)	7.62 (5.05-20.87)	8.07 (5.03-13.26)	8.66 (7.78-31.51)	4.85 (3.95-8.96)	7.32 (4.20-17.83)
IFN-γ (pg/mL)	12.78 (9.27-36.79)	8.75 (6.40-30.92)	13.28 (12.41-15.66)	10.26 (8.17-32.03)	5.43 (4.52-20.64)	14.83 (12.28-21.45)
IL-6/IL-10	1.19 (0.92-1.40)	1.80 (1.45-2.15)	1.15 (0.89-1.47)	1.14 (1.09-1.25)	2.22 (1.44-3.51)	1.30 (1.14-2.77)
IL-8/IL-10	34.52 (3.41-53.38)	88.02 (64.54-96.10)	28.65 (17.59-41.99)	35.10 (11.41-41.25)	93.04 (76.14-99.19)	39.39 (17.43-47.75)
IL-12p70/IL10	1.14 (0.99-1.49)	1.08 (0.90-1.28)	1.78 (0.01-15.66)	1.08 (1.04-1.22)	1.33 (0.70-1.49)	1.74 (0.01-8.49)
TNF-α/IL-10	1.33 (1.13-1.70)	3.09 (1.75-8.92)	1.44 (0.92-3.08)	1.61 (1.46-4.33)	2.19 (1.90-2.47)	1.30 (1.06-2.75)
IFN-γ/IL-10	2.09 (1.53-3.69)	3.36 (2.65-4.56)	2.70 (1.48-3.43)	1.94 (1.38-5.74)	2.86 (2.39-5.04)	3.25 (1.79-3.48)

Note: IL, interleukin; TNF-α, tumor necrosis factor-alpha; IFN-γ, the interferon-gamma.
